# Integration of the expanded disability status scale with ambulation, visual and cognitive tests

**DOI:** 10.1007/s10072-024-07559-9

**Published:** 2024-04-30

**Authors:** Alessio Sarnataro, Nunzia Cuomo, Cinzia Valeria Russo, Antonio Carotenuto, Roberta Lanzillo, Marcello Moccia, Maria Petracca, Vincenzo Brescia Morra, Francesco Saccà

**Affiliations:** Department of Neurosciences and Reproductive and Odontostomatological Sciences, University “Federico II”, Via Pansini, 5, 80131 Naples, Italy

**Keywords:** Disability, Visual, Cognition, Ambulation, Measurement

## Abstract

**Introduction:**

The Expanded Disability Status Scale (EDSS) is usually calculated through a neurological examination with self-reported performance. This may lead to incorrect assessment of Functional System scores (FSs). Aim of our study was to estimate the difference between EDSS obtained during routine visits, or after specific tests.

**Methods:**

We enrolled 670 MS patients that underwent a regular neurology consultation, and visual evaluation using optotype tables, ambulation evaluation with a rodometer, and cognitive assessment with the Brief International Cognitive assessment for MS (BICAMS). We calculated a new integrated EDSS (iEDSS) using the refined values of the FS and compared it to the standard EDSS.

**Results:**

Visual, cerebral and ambulation FSs were significantly higher compared with the self-reported counterpart [+ 1.169 (95%CI 1.077, 1.262; *p* < 0.001), + 0.727 (95%CI 0.653, 0.801; *p* < 0.001) and + 0.822 (95%CI 0.705, 0.939; *p* < 0.001), respectively]. Mean iEDSS was higher than EDSS (+ 0.642; *p* < 0.001). Visual acuity tests worsened the EDSS in 31% of cases, cognitive tests in 10%, ambulation measurement in 35%, all three measurements in 59% of cases.

**Conclusions:**

Objective measurement of FSs results in a more accurate EDSS score in almost two-thirds of cases. This could lead to a more thorough evaluation of patients in the transition or progressive phase.

## Introduction

The Expanded Disability Status Scale (EDSS) is widely used to measure disability of people with multiple sclerosis (MS). The EDSS was developed by Kurtzke in the 1950s and provides a final score determined by Ambulation and seven different Functional Systems (FS): Pyramidal, Cerebellar, Brainstem, Sensory, Bowel/bladder, Visual, Cerebral. The EDSS score ranges from 0 (no disability) to 10 (death due to MS), in 0.5 increments [1–3]. For scores below 4.0, the FS scores alone determine the EDSS. For scores between 4.0 and 6.0, both gait abilities and the FS scores determine the EDSS. Scores above 6.0 depend exclusively on gait and self-care abilities [4].

Most FSs of the EDSS are scored quite accurately through a routine neurological examination. However, Visual and Cerebral FSs, and Ambulation may require additional tests to be scored accurately. This may depend on local practices, as some centers may routinely assess visual abilities, cognition, and ambulation with additional tests, while others rely on self-reported estimates of visual, cognitive, and walking abilities. The risk is that patient’s self-evaluation may result in an over- or under-estimation of the actual abilities.

A previous study examined the difference between self-reported and measured maximum walking distance and found a deep discrepancy, with up to 43.9% of patients erroneously estimating their walking distance [5].

We previously examined the difference between the neurologist’s estimate of cognitive impairment and actual cognition using the Brief International Cognitive Assessment for MS (BICAMS) and orientation tests (OTs) [6]. After performing these objective tests, we calculated the Cerebral FS, and evaluated its impact on final EDSS. We tested 604 MS patients from a different cohort from the one selected for the current study and found that the cognitively enhanced EDSS was different from native EDSS in 16% of all cases. When considering patients with an EDSS ≤ 4.0, the range where FSs play a fundamental role, the proportion of miscalculated EDSS was 25%.

No studies have reported the number of miscalculated visual FSs so far, nor have focused on how this FS is measured during clinical practice in MS centers. At least, it is regularly measured in clinical trials or specific MS cohorts, following specific instructions [7, 8]. Due to the very high number of MS patients worldwide and considering the time constraint of specialized neurologists working in the field at each center, we are confident that visual acuity is currently overlooked during routine practice. This has never been reported in published papers. It is a common understanding between MS centers, that visual acuity with the aid of eye charts, cognition through neuropsychological tests and ambulation with dedicated measurements is not standard clinical practice.

Therefore, an accurate disability assessment is still an unmet need in clinical practice. One solution could be the validation of additional measures, such as manual and cognitive abilities, that could be tested through smartphone and smartwatch-based systems [9–12]. The drawback is that interpretation and decision-making becomes difficult with new measures as clinical trials and Real-World studies have all used the EDSS for disability assessment. A more accurate assessment of the Visual, Cerebral and Ambulation FSs could lead to a better calculation of the EDSS and improve its use in patients’ evaluation.

The aim of the present study was to estimate the difference between FS scores obtained during routine visits, or after specific visual, cognitive and ambulation tests, and to evaluate the impact of this on final EDSS rating.

## Patients and methods

### Study design

This was a single-center study, involving outpatients from our MS center. The study was approved from the local Ethics Committee. Inclusion criteria were a) a confirmed MS diagnosis; b) written informed consent to neuropsychological assessment and collection of clinical variables; We excluded patients on relapse or if a relapse had occurred less than 90 days from study entry. Wheelchair-bound patients were included in the study and ambulation abilities were recorded according to their ability.

We examined patients following clinical practice and calculated a standard EDSS following Neurostatus specifications [4, 7, 8]. Then, patients underwent a detailed and instrumental assessment of the Visual and Cerebral FS, and of Ambulation. The results of the newly estimated FSs were used to determine a new EDSS that we named integrated EDSS (iEDSS). We followed the Neurostatus specifications as well for final EDSS calculations [4, 7, 8]. Neurostatus calculations were performed by neurologists with Neurostatus level C qualification.

### Digital acuity system (DAS) for Visual FS instrumental assessment

Patients underwent a visual evaluation using Landolt's C-target digital eye optotype table through the Smart Optometry App for iPad (Smart Optometry D.O.O., Idrija, Slovenia). Reliability of the app was recently reported and, despite not being suggested for telemedicine, was found to be satisfactory for clinical use [13]. Patients had to wear their habitual correction for the distance (or for the near in case of presbyopia).

Measurement was done monocularly, one eye at a time, at a distance of 40 cm. We used a tape measure for a precise measurement of the distance. This usually corresponded grossly to holding the iPad on the patient’s lap with arms flexed at a 90° angle.

We selected the open circles test for this study. Patients had to choose one of eight possible directions of circle openings. Answers were imputed directly on the iPad screen through a swipe in the direction of the opening itself. In case of motor impairment, an assistant imputed the correct answer for the patient, after answering verbally. The presence of scotoma was assessed based on patient’s reported data.

### Brief international cognitive assessment for MS (BICAMS) study procedures

Tests were administered in a standardized manner, during daytime, in a quiet room, and in a fixed order: Orientation Tests (OTs), Symbol Digit Modalities Test (SDMT), California Verbal Learning Test – II (CVLT-II), Brief Visuospatial Memory Test-Revised (BVMT-R).

Calculation of the Cerebral FS with the BICAMS and OTs occurred as previously reported [6]. Briefly, for every impaired test (i.e. SDMT, CVLT-II, or BVMT-R) the Cerebral FS increased by one point for a maximum score of three. Patients disoriented in one or two spheres (i.e. time, place and person) received a score of four, patients disoriented in all three spheres received a score of five.

### Ambulation measurement

For the Ambulation score, patients were asked to walk through our hospital hallway. They were encouraged to walk without support or using a single or double support as needed. We instructed patients to aim at their best effort and to stop as soon as they were not able to continue for pain, fatigue, or other occurring symptoms. To accurately measure the walking distance, we followed patients with a wheel rodometer (Bosch GWM 32, Gerlingen, Germany). We stopped the test at 1000 m, as additional distance does not result in lower Ambulation scores.

### Statistical analysis

We performed a descriptive analysis of all included variables. We compared scores of single FSs before and after instrumental measurement using a paired *t*-test. The likelihood of an EDSS change after a FS score change was assessed with a logistic regression model, either univariable for the EDSS or multivariable for the iEDSS. For the first model we tested three different models, where the dependent variable was a change in the EDSS score, and independent variables, one at a time, were a change in the Visual, Cerebral and Ambulation FSs. For the multivariable model, the dependent variable was the presence of a change in the EDSS to iEDSS, and the independent variables were changes in Visual, Cerebral and Ambulation FSs that were tested in the same model. We used SPSS version 27.0.1.0 running on MacOS ver. 12.2.

## Results

We enrolled 670 MS patients who underwent both the routine EDSS and the iEDSS assessment. Demographics are shown in Table 1.

**Table 1:** Demographics and current treatment

Variable	Mean ± SD (Range)
Age	44.9 ± 12.7 (16–78)
Age at onset	31.5 ± 11.1 (9–66)
Disease Duration	13.3 ± 10.2 (0–51)
Female gender (n)	440 (66%)
Disease Form	Number (Percentage)
*RR-MS*	541 (83%)
*SP-MS*	78 (11.6%)
*PP-MS*	34 (5.2%)
Current DMT	
*Interferon/Glatiramer Acetate*	154 (23%)
*Teriflunomide*	19 (2.8%)
*Dimethylfumarate*	105 (15.7%)
*Cladribine*	46 (6.9%)
*Fingolimod*	115 (17.2%
*Natalizumab*	79 (11.8%)
*Rituximab*	6 (0.9%)
*Alemtuzumab*	31 (4.6%)
*Ocrelizumab*	84 (12.5%)
*Siponimod*	10 (1.5%)
*No therapy*	21 (3.1%)

*SD* standard deviation, *DMT* disease modifying therapy, *RR-MS* relapsing remitting multiple sclerosis, *SP-MS* secondary progressive multiple sclerosis, *PP-MS* primary progressive multiple sclerosis

**Fig. 1:**
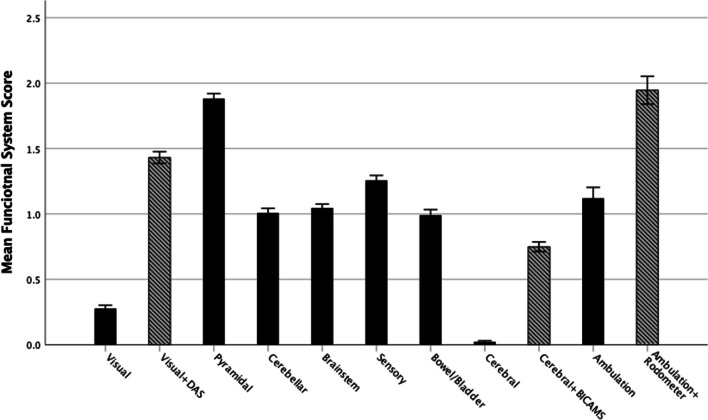
DAS = Digital Acuity System; BICAMS = Brief International Cognitive Assessment for Multiple Sclerosis; *** = *p* < 0.001

Mean and standard deviation of FSs was as follows: Visual 0.28 ± 0.7, Visual + DAS 1.45 ± 1.2, Brainstem 1.05 ± 0.8, Pyramidal 1.90 ± 1.0, Cerebellar 1.00 ± 0.9, Sensory 1.26 ± 1.0, Bowel/Bladder 1.00 ± 1.1, Cerebral 0.02 ± 0.2, Cerebral + Bicams 0.75 ± 0.9, Ambulation 1.15 ± 2.2, Ambulation + Rodometer 2.00 ± 2.8 (Fig. 1).

**Fig. 2:**
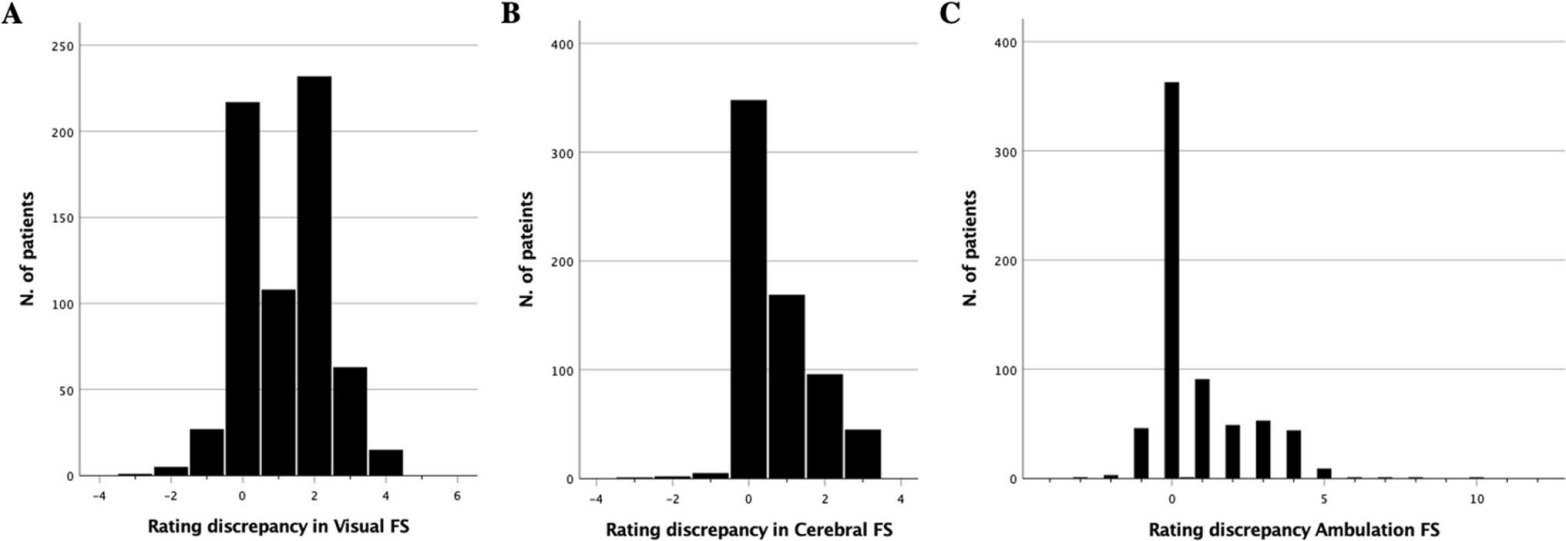
FS = Functional System. **A**) discrepancies between the visual FS as measured through clinical practice and after the use of the digital acuity system; **B**) discrepancies between the cerebral FS as measured through clinical practice and after the integration with neuropsychological tests; **C**) discrepancies between ambulation as measured through clinical practice and after the use of a rodometer and proper testing

Disagreement between the three FS scores, and their instrumentally enhanced version, was very common. The addition of DAS to the Visual FS led to a different score in 453 patients (67.6%), the addition of BICAMS to the Cerebral FS changed the score in 322 patients (48.1%), and the same was true for Ambulation in 307 patients (45.8%). This resulted in different final FS scores: mean difference for Visual FS 1.169 (95% CI 1.077, 1.262; *p* < 0.001), mean difference for Cerebral FS 0.727 (95% CI 0.653, 0.801; *p* < 0.001), mean difference for Ambulation 0.822 (95% CI 0.705,0.939; *p* < 0.001; Fig. 1). The distribution of differences is shown in Fig. 2A-C. Ambulation had the largest disagreement excursion, as expected from the possible scores (i.e. 0–12 vs 0–5 or 0–4), whereas Visual and Cerebral were limited to 1–4 point increase. Visual FS and Ambulation showed a tendency towards a bimodal distribution of the disagreement. The number of disagreements with negative values, i.e. where the instrumentally calculated score was lower than the original, was very low and negligible (Fig. 2A-C).

Mean and standard deviation of EDSS scores were: EDSS 3.09 ± 1.5, EDSS + BICAMS 3.16 ± 1.5, EDSS + DAS 3.27 ± 1.4, EDSS + Rodometer 3.6 ± 1.8, iEDSS 3.7 ± 1.7, while median values were: EDSS 3.0, iEDSS 4.0. This resulted in different final scores compared to the standard EDSS: mean difference for EDSS + BICAMS + 0.07 (95% CI + 0.05, + 0.09; *p* < 0.001), mean difference for EDSS + DAS + 0.18 (95% CI + 0.15, 0.21; *p* < 0.001), mean difference for EDSS + Rodometer + 0.47 (95% CI + 0.40, + 0.54; *p* < 0.001), mean difference for iEDSS + 0.64 (95% CI + 0.58, + 0.71; *p* < 0.001).

**Fig. 3:**
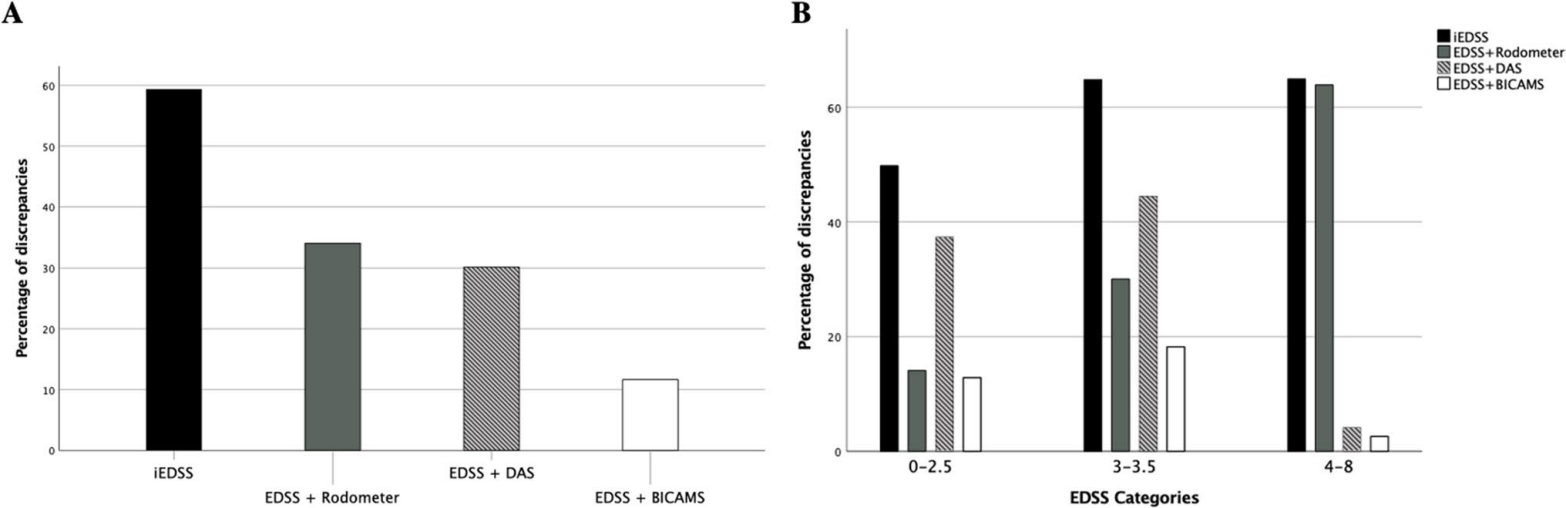
DAS = Digital Acuity System; BICAMS = Brief International Cognitive Assessment for Multiple Sclerosis; EDSS = Expanded Disability Status Scale; iEDSS = integrated EDSS. **A**) Discrepancies in percentage between the standard EDSS calculation and EDSS + BICAMS, EDSS + DAS, EDSS + Rodometer, iEDSS; **B**) Total population was split in three equal parts (EDSS 0–2.5, 3–3.5, 4–8) and discrepancies between standard EDSS, and instrumentally enhanced versions are shown

Instrumental measurement of the Visual FS led to a higher EDSS score in 30.1%, the addition of BICAMS to the Cerebral FS increased the EDSS score in 11.6% of patients, additional estimation of the Ambulation score increased the EDSS in 34% of our patients. The integration of all three measures in the EDSS worsened the original EDSS score in 59.4% of cases (Fig. 3A).

We then divided our population into three groups of equal dimensions based on the EDSS score (i.e. 0–2.5, 3–3.5, 4.0–8.0). The impact of the instrumentally calculated visual and Cerebral FSs was clear for EDSS up to 3.5 and was useless for higher scores (Fig. 3B). In contrast, Rodometer enhanced Ambulation was increasingly useful as the EDSS increased and was the only determinant for scores higher than 3.5.

Patients showing a disagreement between outpatient and instrumentally estimated FSs had an increased likelihood of having a different final EDSS score. Table 2 summarizes the results from the univariable model where a disagreement in a single FS leads to a change in the EDSS, and the multivariable regression model where Visual, Cerebral and Ambulation FSs are independent predictors of a change in the iEDSS.

**Table 2:** Disagreement in FSs for EDSS and iEDSS model

Disagreement in FSs (for each point)	OR	95% CI	*p* values
*Univariable model on EDSS*			
Visual	2.410	2.021, 2.875	< 0.001
Cerebral	4.684	3.479, 6.304	< 0.001
Ambulation	7.493	5.422, 10.354	< 0.001
*Multivariable model on iEDSS*			
Visual	2.526	2.030, 3.144	< 0.001
Cerebral	1.975	1.660, 2.350	< 0.001
Ambulation	1.761	1.420, 2.184	< 0.001

*FS* functional system, *OR* odd’s ratio, *CI* confidence interval, *EDSS* expanded disability status scale, *iEDSS* integrated EDSS

## Discussion

We report on a large cohort of MS patients tested through a routine neurological examination and through additional disability measures aimed at increasing the accuracy of the EDSS. We found that up to 60% of all EDSS scores were higher after combining the results of instrumental Visual, Cognitive and Ambulation assessments.

Visual, Cerebral and Ambulation FSs need additional procedures to accurately measure disability. This is supported by the frequent disagreement between routine and instrumental assessment that was higher for the Visual FS (i.e., 67.6% of all cases), and still problematic for Cerebral (48.1%) and Ambulation (45.8%). Surprisingly, a change in Cerebral FS led to a change in final EDSS score in 11.6% of patients, which was lower than the impact of Visual changes (30.1%) and Ambulation (34%). The reason for a low impact of the Cerebral FS score could be that it is routinely scored at 0, while other FSs have a constant higher rating. Therefore, increasing the Cerebral FS of 1 or 2 points will not worsen the final EDSS. On the other hand, cognitive impairment may occur when other FSs are more impacted, reducing the influence of cognitive measures.

We found a high utility of instrumental measures for low scores of the EDSS (i.e. 0–3.5). In this range, enhanced Visual, Cerebral and Ambulation assessments, all impacted the final score. With scores higher than 3.5, the use of DAS and cognitive measures was negligible (Fig. 3B). This leads to the consideration that for limited resources and time Visual and Cerebral FSs should be instrumentally assessed for lower scores, whereas Ambulation should always be determined with a Rodometer.

Disability measurement has been achieving great importance as new therapies are being available for the progressive phase of the disease. Previous Disease Modifying Therapies were approved for Relapsing–Remitting forms of MS and their goal was to reduce relapses and MRI activity. Since Ocrelizumab and Siponimod were found to be effective in progressive forms of MS [14, 15], an exact measurement of patients’ disability is now a priority for MS centers. This is necessary for both an early diagnosis of secondary progressive MS, and for follow-up purposes during treatment with specific DMTs. For this use, a faulty EDSS, not reflective of the exact disability status, may not capture disease worsening, leaving patients with a stable EDSS and delaying the diagnosis of a progressive phase. The iEDSS improves capturing of precise disability and may thus favor both the diagnosis in a transitioning phase and trigger therapeutic switches. A longitudinal assessment of this cohort with the EDSS and iEDSS will help support the previous statement and the role of additional measurements.

The addition of tests and modification of the EDSS is not new. We reported a BICAMS enhanced version of the EDSS as an attempt to improve the accuracy of the cerebral FS and found that approximately 16% of all calculated EDSS were underestimated, the percentage was 25% when considering EDSS ≤ 4.0 [6]. Morrow et al. reported an improved version of the EDSS using the SDMT and the Fatigue Severity Scale to calculate a modified cerebral FS and found that up to 44% of relapsing patients had a worse improved version of the EDSS [16]. Similarly, Brissart et al. used neuropsychological tests and a fatigue rating scale to better calculate the cerebral FS [17]. Unfortunately the duration of this very precise evaluation could be estimated between 90 to 120 min, making it unapplicable in clinical practice. They showed that one-third of patients with EDSS ≤ 3.5 had an underestimated EDSS.

Apart from a better assessment of single FSs, there is a need for innovative systems able to reduce EDSS inconsistencies and improve final calculation. One example of this is the Neurostatus-eEDSS, which is an electronic tool providing real-time feedback that enables neurologists to correct inconsistencies. Interestingly, during the development of this tool, cerebral FS was the most impacted score by inconsistencies, mainly related to the definition of the score itself and the inclusion of fatigue in the rating [18]. Use of this system in two phase III clinical trials, resulted in 40% of inconsistencies that caused a change in final EDSS scores in 14.8% of assessments [19].

Translating our study into clinical practice poses some challenges, the most evident is the availability of qualified personnel at each MS center. To measure all three FSs one would need a trained neuropsychologist, and at least one nurse/physician performing visual and ambulation tests. We strongly believe that cognitive tests are more reliable if administered in person and not through automated apps as complex procedures are often misunderstood by patients and the risk of having unreliable results is high.

Time is always a serious concern in MS center, and patients’ assessment during this study may have prolonged time needed for each visit. In Autohrs’ experience, the average time to administer the tests was nearly 25 min overall. Future addition of DAS to clinical practice may be helpful as it does not require assistance, except for few selected patients, in whom physical disability interferes with the iPad use. For many patients, results can be automatically collected and stored. Also, ambulation can be measured through ambient measurement systems that can output additional quantitative and qualitative data in an automated way (e.g. 25-foot walk test) [20]. Both DAS and ambulation assessment could be done in the waiting room before neurological assessment and result in time optimization.

Results from these enhanced measures could be automatically sent to each center’s electronic database. As soon as all other FS scores, after a routine visit, are uploaded in the system, calculation of the iEDSS could take place instantly. To anticipate this opportunity, we have created and validated an iPad-based version of the BICAMS and integrated it in our center’s database. This allows for an instant upload of the Cerebral FS after a brief neuropsychological evaluation [21].

In conclusion, we think that our study supports the need for a better calculation of the EDSS through an improved assessment of the Visual, Cerebral and Ambulation FSs. A future follow-up on our RR-MS patients will help us understand if the iEDSS can allow for early SP-MS diagnosis and prompter therapeutic switch. Also, integration of the iEDSS with automated feedback, such as the Neurostatus-eEDSS, could help further reduce inconsistencies and lead to a more credible result.

## Declarations

Alessio Sarnataro has nothing to disclose. Nunzia Cuomo has nothing to disclose. Cinzia Valeria Russo has nothing to disclose. Antonio Carotenuto received research grants from ALMIRALL, and honoraria from Novartis, Merck, and Biogen. Roberta Lanzillo received personal compensation for speaking or consultancy from Biogen, Teva, Genzyme, Merck, Novartis, and Almirall. Marcello Moccia has received research grants from ECTRIMS-MAGNIMS, UK MS Society, and Merck; honoraria from Biogen, Celgene, Merck, Roche, and Sanofi Genzyme. Maria Petracca discloses travel/meeting expenses from Novartis, Roche and Merck, speaking honoraria from HEALTH&LIFE S.r.l., honoraria for consulting services from Biogen and research grants from Baroni Foundation. Vincenzo Brescia Morra received personal compensation for speaking or consultancy from Biogen, Teva, Genzyme, Merck, Novartis, and Almirall. Francesco Saccà received public speaking honoraria from Alexion, Argenx, Biogen, Mylan, Novartis, Roche, Sanofi, Teva; he also received compensation for Advisory boards or consultation fees from Alexion, Almirall, Argenx, Avexis, Biogen, Forward Pharma, Lexeo Therapeutics, Merk, Novartis, Novatek, Roche, Sanofi, Takeda; he is principal investigator in trials for Alexion, Argenx, Immunovant, Novartis, Prilenia.
